# Particular characteristics of allergic symptoms in tropical environments: follow up to 24 months in the FRAAT birth cohort study

**DOI:** 10.1186/1471-2466-12-13

**Published:** 2012-03-22

**Authors:** Nathalie Acevedo, Jorge Sánchez, Josefina Zakzuk, Adriana Bornacelly, Carlos Quiróz, Álvaro Alvarez, Marta Puello, Ketty Mendoza, Dalgys Martínez, Dilia Mercado, Silvia Jiménez, Luis Caraballo

**Affiliations:** 1 Institute for Immunological Research, University of Cartagena, Cartagena, Colombia; 2 Foundation for the Development of Medical and Biological Sciences (Fundemeb), Cartagena, Colombia; 3 Department of Microbiology, Faculty of Medicine, University of Cartagena, Cartagena, Colombia

**Keywords:** Birth cohort study, Wheezing, Allergy, Asthma, Rhinitis, Eczema, Atopic march, The tropics, Parasite, Poverty, Cartagena, Latin America

## Abstract

**Background:**

Early wheezing and asthma are relevant health problems in the tropics. Mite sensitization is an important risk factor, but the roles of others, inherent in poverty, are unknown. We designed a birth-cohort study in Cartagena (Colombia) to investigate genetic and environmental risk factors for asthma and atopy, considering as particular features perennial exposure to mites, parasite infections and poor living conditions.

**Methods:**

Pregnant women representative of the low-income suburbs of the city were randomly screened for eligibility at delivery; 326 mother-infant pairs were included at baseline and biological samples were collected from birth to 24 months for immunological testing, molecular genetics and gene expression analysis. Pre and post-natal information was collected using questionnaires.

**Results:**

94% of families were from the poorest communes of the city, 40% lacked sewage and 11% tap-water. Intestinal parasites were found as early as 3 months; by the second year, 37.9% of children have had parasites and 5.22% detectable eggs of *Ascaris lumbricoides *in stools (Median 3458 epg, IQR 975-9256). The prevalence of "wheezing ever" was 17.5% at 6 months, 31.1% at 12 months and 38.3% at 24 months; and recurrent wheezing (3 or more episodes) 7.1% at 12 months and 14.2% at 24 months. Maternal rhinitis [aOR 3.03 (95%CI 1.60-5.74), *p = *0.001] and male gender [aOR 2.09 (95%CI 1.09 - 4.01), *p = *0.026], increased risk for wheezing at 6 months. At 24 months, maternal asthma was the main predisposing factor for wheezing [aOR 3.65 (95%CI 1.23-10.8), *p = *0.01]. Clinical symptoms of milk/egg allergy or other food-induced allergies were scarce (1.8%) and no case of atopic eczema was observed.

**Conclusions:**

Wheezing is the most frequent phenotype during the first 24 months of life and is strongly associated with maternal asthma. At 24 months, the natural history of allergic symptoms is different to the "atopic march" described in some industrialized countries. This cohort is representative of socially deprived urban areas of underdeveloped tropical countries. The collection of biological samples, data on exposure and defined phenotypes, will contribute to understand the gene/environment interactions leading to allergy inception and evolution.

## Background

**Figure 1:**
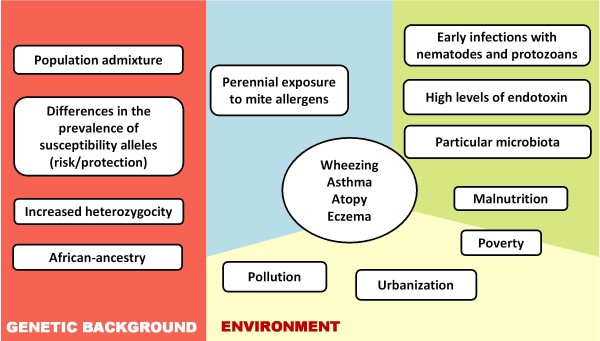
**Gene-environment interactions and the susceptibility to allergic diseases in tropical underdeveloped regions**. A summary of risk factors that may influence the inception of wheezing and other allergic phenotypes in socially deprived urban areas of the tropics. Some may at first instance seem to be protective; however the rates of allergy and asthma in urbanized-areas of South America indicate the contrary.

The causes of asthma and other allergic diseases remain unknown. Genetic and epidemiological studies suggest that for these multifactorial diseases the expression of different phenotypes depend on complex interactions between susceptibility genes and the environment [1]. This is reflected in the wide differences in prevalence and natural history of allergic diseases around the world [2]. In many regions of Latin America asthma is a public health problem affecting children and adolescents in urban areas; wheezing, asthma and allergic rhinitis are very frequent in some regions [3], with rates similar or even higher than in industrialized countries [4,5]. Interestingly, anticipated protective factors, such as low hygienic conditions, do not confer protection in poor and overcrowded communities, where a high prevalence of asthma and early infections concur [6-8]. In addition, there are disparities among some phenotypes, such as allergic sensitization and prevalence of atopic eczema, when compared to those observed in industrialized countries [9,10]. In urban zones of Colombia, asthma is the most common chronic disease in children and IgE sensitization to mites is a hallmark in most patients [11-13]. The population of Cartagena, Colombia, has been previously studied to investigate genetic and environmental risk factors for asthma and allergy, not only because asthma is common [14] but particularly because the city is in a tropical region where a warm and humid environment, facilitate the growth of a diverse mite fauna and the perennial exposure to high concentrations of their allergens [15-17]. Moreover, most of the population is poor and exposed to parasites, generating an interesting setting to study the influence of environmental factors on the susceptibility to allergic diseases (Figure 1).

There are few Latin American birth-cohort focused on allergic diseases [18-20] and they have explored several phenotypes and risk factors using different study designs. For example, Lopez et al. detected sensitization to *D. pteronyssinus *in 30% of wheezing and 11% of asymptomatic infants at 12 months of age in a prospective study (n = 102) in Brazil. They also found a weak association between wheezing and specific IgE to mites at 12 months. Environmental exposures and socioeconomic status were not evaluated [18]. Rullo et al. studied the role of respiratory infections, exposure to mouse allergens and breastfeeding on wheezing in 104 children living in a socially deprived community of Brazil. Analysis at 30 months showed strong association of wheezing with "respiratory infections requiring antibiotics". No association was found with endotoxin exposure or mite sensitization [19]. Cooper et al. in a study protocol presented the strategies for investigating the impact of early life exposure to geohelminth infections on the development of vaccine immunity, allergic sensitization and allergic inflammatory diseases in 2,403 neonates followed up to 8 years of age [20]. Our study population is an urban low-income community of admixed genetic background [21,22], living in the tropics under limited sanitary conditions and exposed to mites and helminth allergens. We hypothesize that, for children growing up under these particular genetic and environmental conditions, the prevalence of some allergic phenotypes, as well as the nature and effects of risk factors are different to those found in cohorts from industrialized countries.

The aims of this study were: 1. To create a community-based birth cohort study for analyzing the effects of allergen exposure, early parasite infections and poor living conditions on the inception of allergic diseases, specially asthma; 2. To evaluate the effects of prenatal and other risk factors on the prevalence of wheezing and eczema and 3. To prospectively collect biological samples of children living in poor neighborhoods of a tropical city for further immunological testing, molecular genetics and molecular microbiology screenings. Here we describe the study protocol, baseline characteristics, demographical observations and risk factors for wheezing up to 24 months, of the "Risk Factors for Asthma and Allergy in the Tropics" (FRAAT) study.

## Methods

### Design, location and study population

The Ethic Committee of the "Fundación Santa Fe de Bogotá", Bogotá-Colombia, approved this study (CCEI-282-206). We created a community based birth cohort for a prospective follow up and collection of epidemiological data and biological samples. Cartagena is a tropical city in the Caribbean North Coast of Colombia (10° 23' 59″ North, 75° 30' 52″ West) with an average annual temperature of 28°C and 80% of relative humidity. Most inhabitants are poor according to governmental indexes that assess type of housing, overcrowding (three or more people per bedroom), access to basic services, income and school attendance. This socioeconomic stratification ranges from 1 to 6 and 90% of the population is grouped in the lowest strata, 1 to 3 [23]. The majority of study participants belonged to the poorest communes and shared environmental conditions. The genetic background of this population resulted from racial admixture between Native Americans, Spaniards, and an important proportion (37.9%) of African ancestry [22].

### Eligibility criteria and enrollment procedures

To ensure adequate representation of the city population, pregnant women attending two public medical centers during parturition (Clínica Maternidad Rafael Calvo and Centro de Atención Permanente La Candelaria) were screened for eligibility by physicians of the research staff between August 2007 and May 2008. These centers serve the majority of the lowest social strata in the city. Mothers were interrogated during admission to the delivery room, examined and followed during labor. Only those fulfilling the following criteria were included: healthy women natives from Cartagena or residents in the city for at least 5 years prior to pregnancy, with singleton pregnancy, without obstetric complications and/or chronic diseases. The exclusion criteria were: high-risk pregnancy, pre-eclampsia, dystocia, autoimmune diseases, tumors, or current use of oral steroids, although asthma was not an exclusion criterion. Informed consent for participating in the study and collecting a cord blood sample was obtained from each mother before delivery. After birth, newborns were examined and those fulfilling the following criteria were included: product of low-risk pregnancy, born by vaginal delivery with a gestational age between 37 and 42 weeks according to Lubchenco method [24], without labor complications, weight > 2500 grams and APGAR score of at least 7 at five minutes after birth. Children born prematurely, requiring reanimation, pulmonary maturation with steroids, management in intensive care unit after delivery or having congenital deformities were excluded. Three hundred and twenty six mothers agreed to participate with their child and enrolled the study at baseline. After delivery, all families received an explanation about the investigation and signed a written informed consent to participate.

### Collection of baseline data and follow-up

**Figure 2:**
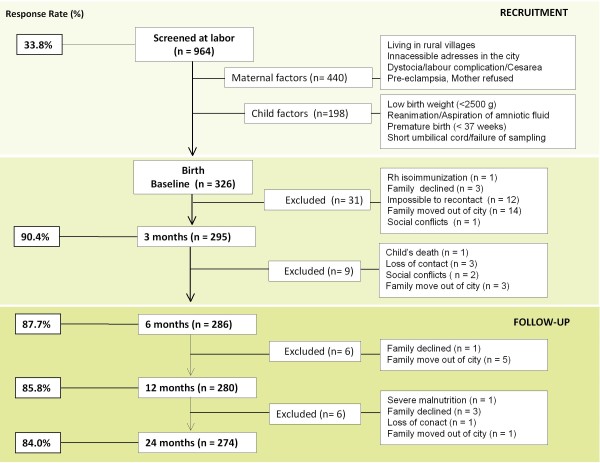
**Flow-chart of data collection and response rates during the first 24 months in the FRAAT study**. Responses rates (left boxes) are shown as a percentage, relative to the total number of screened mothers during recruitment and to the baseline sample size before and during follow up. Exclusion criteria, applied during recruitment, and drop-out causes (right boxes) are also detailed.

**Figure 3:**
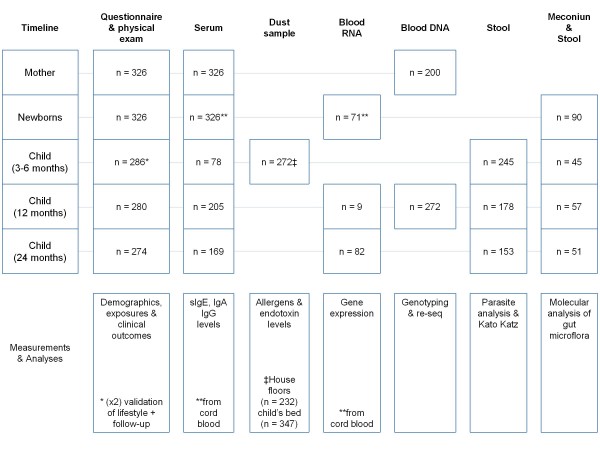
**Timeline of data and sample collection from 0 to 24 months**.

Questionnaires were based on the written Spanish version of the International Study of Asthma and Allergies in Childhood, ISAAC [25] and others tested in our population [9,14]. We added questions to cover particular factors regarding living conditions and poverty indicators. These questions were tested previously for sensitivity and specificity in a sub-study of 97 random-selected women attending the aforementioned public centers, using as gold standard the direct observation of homes by the investigators during domiciliary visits. For those variables related with maternal factors (such as parity, education, socioeconomic stratum) the mean sensitivity was 94% and mean specificity 92%. For living conditions and exposures the results were variable, some questions had good sensitivity and specificity (e.g., sewage system), while others had higher sensitivity than specificity (tap water) and vice versa (trash burning). In cases of disagreements of answers about a particular variable, the information taken by the investigators during domiciliary visits was used for analysis. The baseline questionnaire addressing prenatal risk factors was administered in the hospital within 24 hours post-partum by a physician of the research staff interviewing the mother, with input from relatives on the questions about family history of allergic disease, parental lineage, etc.. The variables included antecedents of asthma and allergic diseases in the mother, father and relatives, socioeconomic status (monthly income, occupation, social stratum), smoking during pregnancy or passive exposure to cigarette smoke, exposure to fume from trash burning, type of domestic cooking fuel (electric, natural gas, charcoal or firewood); location of the house regarding industrial area and proximity to high-traffic/main roads, pet ownership and duration of pet contact, building materials of houses, type of floor (tile, bare concrete or soil), access to tap water and sewage system, dietary control during pregnancy and antibiotic use. The second questionnaire was administered by a physician of the research staff during a domiciliary visit; it was designed to confirm the information about the houses, the sociodemographic indicators and self-reported exposures. Due to logistic reasons (mainly difficulties to find home addresses), this was done within 3 to 6 months after birth. The follow up was performed through the outpatient service at the Institute for Immunological Research, where mothers regularly attended for child's controls or during domiciliary visits. Follow up to 24 months was completed in 84% of the children (Figure 2). When performed, the medical evaluations through 3, 6, 12 and 24 months, included a post-natal questionnaire, physical examination and collection of biological samples (Figure 3).

### Blood samples

A cord blood sample was obtained from the maternal portion immediately after delivery. To avoid contamination with maternal blood, the distal portion of the umbilical cord was closed, the cord was thoroughly cleaned with ethanol and the umbilical vein punctured with disposable syringe. The blood was collected in sterile 15 mL tubes and transported to the laboratory, where serum was separated and frozen for future serological tests. Blood samples from mothers were taken by venipuncture within 12 hours after delivery using the appropriate tubes to obtain sera for antibody determinations and buffy coat as a source of maternal DNA. In children, blood samples were obtained between 6 and 24 months (Figure 3) by venipuncture using disposable syringe. Sera were stored at -20°C for antibody determinations and the clot as a source of DNA. Among the serological test are total IgE and specific IgE to: *Dermatophagoides pteronyssinus, Blomia tropicalis, Ascaris spp.*, purified Ascaris allergens ABA-1 and Asc l 3, cockroach, egg, cat epithelia, mouse, cow's milk and *Staphylococcus aureus *endotoxin (SEB). Also, other parameters of potential value as risk factors, such as vitamin D, will be evaluated. For RNA collection, 3 mL of cord blood and infant blood were immediately added to PAXgene Blood tubes (PreAnalytix Cat. 762165). RNA was extracted using the PAXgene Blood RNA kit (QIAGEN, Cat. 762134) and kept frozen at -80°C.

### Stool samples and parasitological examination

When possible, a meconium sample was collected after delivery in sterile 1.5 mL tubes and kept at -20°C. Mothers were provided with sterile containers and instructed to collect stool samples from their children at different time points between 3 and 24 months. Parasitological analyses were done using 0.85% saline solution and Lugol staining. *Ascaris lumbricoides *egg counts were done by the Kato Katz method using a commercial kit (Copro Kit, C&M Medical, Brazil) and the results expressed as egg per gram of feces (epg). The presence of eggs from geohelminths or parasite visualization were considered diagnostics of active infection. In order to analyze the gut microbiota using molecular tools, DNA was extracted from meconium and stool samples (QIAamp DNA stool Mini kit, QIAGEN, Cat. 515004) following the manufacturer instructions and stored at -80°C.

### Collection of dust samples

For measuring allergen and endotoxin levels, house-dust samples were collected from children homes at 6 months, using a vacuum cleaner (Cyking V-CA241HT, LG Electronics, Korea) adapted to 25 μM cellulose filters. Children mattress and bedroom floor were aspirated two minutes over 1 m^2 ^area as described [26]. Dust samples were placed in sterile recipients, weighted and extracted in 2 mL of pyrogen-free water-0.05% Tween 20 per 100 grams of fine dust, mixed during 1 hour at room temperature and centrifuged (3000 g, 4°C, 10 minutes). An aliquot of supernatant (1/10 of the total volume) was kept at -20°C for endotoxin quantification. The substracted volume was replaced with PBS 10 × -0.05% Tween 20 10 mM PMSF and mixed overnight for protein extraction. After centrifugation aliquots for allergen quantification were obtained and kept at -20°C.

### Assessment and validation of clinical outcomes

Wheezing was defined as expiratory stridor with shortness of breath or whistling on children chest. Mothers were interrogated on the question "Have your child ever had wheezing or whistling in the chest at any time in the past?" For each affirmative response additional validation questions were done to obtain further information about the episode, medications and medical management. Only cases with documented medical diagnosis during visits to health centers and/or wheeze detected during physical examination by the research staff, were considered as wheezing episodes. Based on the number of wheezing between 0 and 24 months, children were classified as: Never wheezers (no wheezing at any time point), occasional wheezers (1-2 episodes) or recurrent wheezers (3 or more episodes in 12 months). Eczema was diagnosed when all the following criteria were present: (1) evidence of itchy skin/pruritus, (2) visible flexural dermatitis, (3) typical morphology and distribution including facial, neck and extensor involvement and (4) dry skin, based on Williams [27-29] after Hanifin [30]. We have used these modified criteria considering that in this community other causes of "itchy skin" are common (scabies, insect stings, helminth induced rashes etc); for the same reason the diagnosis always included medical examination. All children whose mothers referred having itching for more than 2 weeks were re-examined every 3 months during 12 months.

The diagnosis of asthma and other allergic diseases among mothers was done using a questionnaire, [9] and physical examination by a physician from the research staff. Atopy was defined as a positive immediate response to one or more allergens in the skin prick tests, performed between 6 and 12 months, during children follow up. The test was considered positive if the mean diameter of the wheal at 15 minutes was > 3 mm than the negative control. Mothers were tested with a set of aeroallergens, including mites (*D. pteronyssinus*, *B. tropicalis, Aleuroglyphus ovatus, Chortoglyphus arcuatus, Lepidoglyphus destructor, Suidasia medanensis*), cockroach (*Periplaneta Americana*), pollen/grass (*Betula alba, Phleum pretense, Artemisia*), pets (dog and cat), and molds (*Alternaria alternata*, *Penicillium crysogenum*, *Aspergillus fumigatus*), kindly supplied by Leti, Spain. Histamine phosphate [10 mg/mL] was used as positive control and glycerol as negative control. Children will be skin tested after the second year of age.

### Data analysis

Information from interviews was recorded on paper forms, reviewed for accuracy and completeness, and then entered into the database. Statistical analyses were done using SPSS v.13.0 (Chicago, IL, USA). Frequencies and descriptive statistics were calculated at baseline, 6, 12 and 24 months. Chi-square was used to analyze the differences between proportions. For contingency tables with less than 10 cases in any cell, the Fisher's exact test was used. To analyze which parental and/or prenatal factors were related to the development of wheezing, multivariate analyses were performed with children having complete exposures data at 6, 12 and 24 months. Crude odds ratios (OR) and 95% confidence interval were calculated. For risk factors having a significance level p ≤ 0.05, adjusted odds ratios (aORs) and 95% confidence intervals (CIs) were obtained using binary logistic and multinomial logistic regression. Covariates were introduced in the final model if their inclusion changed the estimate of the crude OR by more than 10%. The outcome (dependent) variables were wheezing ever and recurrent wheezing at 6, 12 and 24 months. Variables were analyzed as categorical (e.g. maternal asthma, yes/no) or continuous (e.g. maternal age, number of siblings, birth weight).

### Sample size

We aimed to have at least 100 wheezers with prospective sampling for immunological and molecular screenings up to 24 months. Based on the national prevalence rates [9,14], we estimated that a minimum sample of 400 children will give us the possibility to recruit that number of cases. For association studies, power was calculated assuming an independent case-control design and expressing the alternative hypothesis as Odds Ratio (ψ). For a given risk factor the calculation is done by setting type I error probability at α = 0.05, number of wheezing cases (n), probability of exposure among non-wheezers (ρ0), and the ratio of controls/cases (m) [31].

## Results

### Demographic characteristics of the population

Three hundred and twenty six mother-infants pairs were included. Fifty-two families were lost during follow-up (Figure 2). Reasons for exclusion were: moving out of city to rural villages (n = 23), lost of contact by inaccessible addresses or lack of telephones (n = 16), family declined (n = 7), social conflicts (n = 3), child dead (n = 1), severe malnutrition (n = 1) and Rh isoinmunization (n = 1). Most families were lost in the interval of 0 to 6 months (n = 40), afterwards 12 families drop-out between 7 and 24 months. The sociodemographic characteristics of the excluded families were similar to those that continued in the study. Antecedents of allergic diseases were similar between excluded and non-excluded mothers and did not influence the willingness to participate. Maternal characteristics and pre-natal exposures to risk factors are summarized in Table 1. All families had similar environmental and living conditions. Ninety four percent of participants were clustered in the urban area of the poorest communes ("Comunas") of Cartagena and the rest in two rural villages, La Boquilla (3.1%, n = 10) and Pasacaballos (2.8%, n = 9). The ethnicity of the population was homogeneous, 74.8% of mothers were born in Cartagena, 24.6% in rural villages of the Northwest Coast of Colombia and 0.2% in the inner country. Most of the mothers were young (mean maternal age ± standard deviation, 23.2 ± 5.8 years), 58.9% multiparous, 59.5% house-wives and 62.5 with low level of education. Regarding prenatal exposures, 97.5% denied having smoked during pregnancy but 43.6% reported intradomiciliary secondary-exposure to a median of 35 cigarettes per week (IQR 14 - 70) and 1.36 ± 0.68 (mean ± standard deviation) smokers per household. Some families had habitual contact with fume from cooking with firewood and/or trash burning at homes or their neighborhoods. Intradomiciliary exposure to pets during pregnancy was reported in 48.2% of mothers, being dogs the most common, and to poultry and pigs in 13.5% (Table 1). The prevalence of allergic diseases in mothers is presented in Table 2. Asthma and allergic rhinitis were the most common diseases and dust mite allergens the main sensitizers. Interestingly, cases of atopic eczema were not observed in mothers.

**Table 1:** Demographic characteristics of mothers and prevalence of prenatal exposures

		**Baseline**	**24 months (n = 274)**	
		
**Factor**		**(n = 326)**	**non wheezers** **(n = 169)**	**wheezers** **(n = 105)**
Maternal age at child birth [n (%)]				
	14-18	80 (24.5)	39 (23.1)	27 (25.7)
	19-24	139 (42.6)	68 (40.2)	50 (47.6)
	25-30	67 (20.6)	36 (21.3)	19 (18.1)
	31-35	22 (6.7)	15 (8.9)	5 (4.8)
	36-42	18 (5.5)	11 (6.5)	4 (3.8)
Maternal age (Mean ± SD)		23.2 ± 5.83	23.6 ± 6.15	22.5 ± 5.37
Maternal education				
	None	5 (1.5)	2 (1.2)	2 (1.9)
	Primary school only	57 (17.4)	28 (16.5)	15 (14.3)
	High school incomplete	142 (43.6)	69 (40.8)	48 (45.7)
	High school complete	72 (22.1)	42 (24.9)	23 (21.9)
	Technical studies	46 (14.1)	26 (15.4)	15 (14.3)
	University	4 (1.2)	2 (1.2)	2 (1.9)
Parity	Primipara	134 (41.1)	73 (43.2)	41 (39.0)
No. of other children (Mean ± SD)		1.17 ± 1.35	1.14 ± 1.32	1.19 ± 1.31
Socioeconomic stratum				
	1 (poorest)	257 (78.8)	135 (79.9)	79 (75.2)
	2	59 (18.1)	29 (17.2)	22 (21.0)
	3	10 (3.1)	5 (3.0)	4 (3.8)
Type of house				
	Brick	243 (74.5)	127 (75.1)	79 (75.2)
	Wood	83 (25.5)	42 (24.9)	26 (24.8)
Type of house floor				
	Tile	90 (27.6)	49 (29.0)	28 (26.7)
	Bare concrete	172 (52.7)	87 (51.5)	58 (55.2)
	Soil	64 (19.6)	33 (19.5)	19 (18.1)
Tap water	Yes	290 (89)	147 (87.0)	95 (90.5)
Sewage system	Yes	191 (58.6)	101 (59.8)	61 (58.1)
Exposure to pests at home*				
	Cockroaches	255 (78.2)	137 (81.1)	80 (76.2)
	Rodents (mice, rats)	275 (84.4)	139 (82.2)	92 (87.6)
Smoking and contaminants during pregnancy				
	Maternal smoking (ever)	16 (4.9)	9 (5.3)	3 (2.9)
	Maternal smoking during pregnancy	8 (2.5)	5 (3.0)	2 (1.9)
	Intradomiciliary passive exposure	142 (43.6)	67 (39.6)	48 (45.7)
Type of domestic cooking fuel				
	Wood/Coal	39 (12)	19 (11.2)	13 (12.4)
	Propane	69 (21.2)	36 (21.3)	20 (19.0)
	Natural Gas	244 (74.8)	124 (73.3)	82 (78.0)
	Electricity	3 (0.9)	2 (1.18)	1 (0.96)
Trash burning at home during pregnancy	Yes	65 (19.9)	34 (20.1)	22 (21.0)
Passive exposure to trash fume at neighborhood	Yes	172 (52.8)	88 (52.1)	60 (57.1)
Exposure to pets and to other domestic animals				
	Living with pet during pregnancy	157 (48.2)	82 (48.5)	52 (49.5)
	Dog during pregnancy	143 (43.9)	77 (45.6)	48 (45.7)
	Cat during pregnancy	41 (12.6)	23 (13.6)	10 (9.5)
Living with dog/cat during the whole pregnancy	Yes	107 (32.8)	55 (32.5)	37 (35.2)
Intradomiciliary contact poultry/pigs during pregnancy	Yes	44 (13.5)	20 (11.8)	17 (16.2)
Antibiotics during pregnancy				
	Yes	159 (48.7)**	80 (47.3)	57 (54.2)
	None	157 (48.1)	84 (49.7)	46 (43.8)
	Missing/no remember	10 (3.06)	5 (2.95)	2 (1.90)

* As identified by the families by sightseeing and employing active methods of eradication

** The most frequent cause was urinary tract infection. The most frequent antibiotics were ampicillin (32%), cephalosporin (11.9%), amoxicillin (10.6%) and metronidazole (9.4%).

SD: Standard deviation

**Table 2:** Maternal antecedents of allergic diseases (n = 326)

Phenotype	n (%)	
Current asthma	21 (6.44)^†^	
Duration of asthma (Mean, SD)	14.0 ± 9.01 years	
Current rhinitis	84 (25.7)^‡^	
Eczema	0 (0)	
Food allergy	23 (7.1)	
Drug allergy	5 (1.5)	
Family history of asthma*	53 (16.3)	
Allergic sensitization (n = 265)**	**n (%)**	Any allergy symptom (%)***
Atopy (at least 1 positive test)	93 (35)	(50.5)
Mite sensitized	82 (30.9)	(54.8)
*D. pteronyssinus*	59 (22.3)	(59.3)
*B. tropicalis*	61 (23.0)	(54)
Cockroach	12 (4.5)	(58)
Cat	7 (2.6)	(42)
Dog	7 (2.6)	(57)
Molds (Pen, Asp, Alt)	8 (3.0)	(100)
Pollens (Art, Phl, Bet, Acacia)	7 (2.6)	(57)

†Probability of exposure in non-wheezers (ρ_0 _= 0.05)

‡Probability of exposure in non-wheezers (ρ_0 = _0.26)

*Asthma in parents, grand-parents and/or siblings

** As defined by skin tests

*** Asthma, rhinitis, reported food allergy

SD: Standard deviation

### Living conditions of children and particular environmental exposures

Children were visited at home by the research staff to investigate risk factors and to validate the information collected in baseline questionnaires. Post-natal sociodemographic conditions were similar to those described at baseline. The infant group included 139 females (48.6%) and 147 males (51.4%); most of them living in brick houses with floors of bare concrete, in non-paved streets. Forty per cent lacked sewage system, 20% toilettes and 11% tap water. Usually there were 6 people per household and most children lived with their parents, grand-parents, siblings and other relatives; 35% of them overcrowded, sharing bedrooms and mattresses with parents and older siblings. Furthermore, 35% of families had no fridge and 54% lacked sink for dishwashing. Early infections with parasites were detected as early as 3 months of age. The most common parasites were of the genera *Entamoeba ssp*. Among nematodes *Ascaris lumbricoides *was the most frequent, affecting 2.71% of children at 12 months and 5.22% at 24 months (Table 3). By 12 months, the median egg counts of *A. lumbricoides *were low (78 epg, IQR 76-1452) but increased between the first and the second year (3458 epg, IQR 975-9256), suggesting that in this age range *A. lumbricoides *infections are of low intensity (> 90% of subjects with egg counts < 50.000 epg) [32]. In addition, 101 had received anti-parasitic treatment and 95% at least one cycle of antibiotics (mean age of the first treatment 2 ± 3.4 months), due in part to the high prevalence of infectious diseases, e.g. pneumonia, urinary tract infections, bacillary dysentery.

**Table 3:** Prevalence of parasitic infection as determined by stool examination

	0-6 months (n = 200)	0-12 months (n = 258)	13-24 months (n = 153)
**Any parasite**	17 (8.5%)	62 (21.3%)	58 (37.9%)
**Polyparasitism**	1 (0.5%)	2 (1.13%)	9 (5.88%)
**Protoozoan**			
*Entamoeba ssp*.	12 (6%)	34 (13.1%)	31 (20.2%)
*Giardia lamblia*	1 (0.5%)	12 (4.65%)	18 (11.7%)
*Blastocystis hominis*	1 (0.5%)	3 (1.16%)	3 (1.96%)
*Endolinax nana*	0 (0%)	1(0.38)	2 (1.30%)
*Balantidium coli*	0 (0%)	0 (0%)	1 (0.65%)
**Helminths**			
*Ascaris lumbricoides*	2 (1%)	7 (2.71%)	8 (5.22%)
*Trichuris trichiura*	0 (0%)	2 (0.77%)	2 (1.30%)
*Ancylostoma duodenalis*	2 (1%)	3 (1.16%)	3 (1.96%)
*Strongyloides stercolaris*	0 (0%)	0 (0%)	1 (0.65%)

### Prevalence of wheezing and atopic eczema

The prevalence of "wheezing ever" was 17.5% at 6 months, 31.1% at 12 months and 38.3% at 24 months. Recurrent wheezing (3 or more episodes) was present in 7.1% of children at 12 months and 14.2% at 24 months (Table 4). As confirmed by questionnaires and medical records, 94% of children wheezing between 0 and 24 months attended a medical center or emergency room at least once; 84% received salbutamol, 76% antihistaminics and 43% oral steroids. Hospitalizations were documented in 23% of cases. In addition, half of children wheezing between 0 and 6 months continued wheezing until 24 months. At 24 months, 18.8% of children had experienced at least one episode of skin rash/hives, 11.8% of them treated with antihistamines by a physician. In 7.4% of cases no potential inducer could be identified. Drugs were commonly incriminated (6.9%), including antibiotics (amoxicillin, ampicillin) and anti-inflammatories (metamizole, ibuprofen). In 1.8% of cases the symptoms were related to food ingestion. Only two cases of rash/hives induced by egg ingestion and two cases by milk were clinically documented by the staff. According to our diagnostic criteria, no case of eczema was detected between 0 and 24 months.

**Table 4:** Frequency of wheezing among children from 0 to 24 months

	0-6 months (n = 286)	7-12 months (n = 280)	13-24 months (n = 274)	Cumulative **0**-**12 months** (n = 280)	Cumulative **0**-**24 months** (n = 274)
Wheezers (n)	50	59	64	87	105
Non-wheezers (n)	236	221	210	193	169
Prevalence of wheeze (%)	17.5	21.1	23.4	31.1	38.3
Occasional wheezers (1 or 2 episodes)	-	-	-	67 (23.9%)	66 (24.1%)
Recurrent wheezers (3 or more episodes)	-	-	-	20 (7.1%)	39 (14.2%)
Children that only wheeze at time interval (n)	21	17	19	**-**	**-**
Number of children that wheeze for the first time at each time interval	50	37	19	**-**	**-**

### Risk factors for wheezing during the first two years

We analyzed the effects of prenatal sociodemographic characteristics and environmental exposures on the risk of wheezing at 6, 12 and 24 months and/or recurrent wheezing during the first 24 months (Table 5). Maternal-allergic traits were the most important predictors of children's susceptibility during the first two years of life. Maternal allergic rhinitis was associated with wheezing ever at 6 months and this association held up after adjustment for gender [aOR 3.03 (95%CI 1.60-5.74), *p = *0.001]. Male gender was associated with increased susceptibility of wheezing during the interval 0 to 6 months, and the effect was independent of maternal rhinitis [aOR 2.09 (95%CI 1.09 - 4.01), *p = *0.026]. There was association between maternal asthma and increased risk of wheezing between 7 and 12 months, maintained after adjustment by maternal age and child gender [aOR 3.87 (95%CI 1.24-12.1), *p = *0.02]. When pooling all wheezing cases during 0 to 12 months (n = 87), maternal asthma was associated with wheezing [aOR 3.48 (95%CI, 1.27-9.54), *p = *0.015], but the effect of gender was no longer significant [aOR 1.2 (95%CI, 0.95-2.1), *p = *0.07]. Maternal asthma was the only prenatal factor associated with wheezing at 24 months [aOR 3.65 (95%CI 1.23-10.8) *p = *0.01], after adjustment by maternal age, number of siblings and child birth weight and gender. Moreover, it was associated with recurrent wheezing (Table 5), with significant effects after adjustment for maternal age, number of siblings and birth weight [aOR 4.42 (95%CI 1.46-13.4), *p = *0.008]. Maternal atopy in the absence of asthma was not associated with wheezing at 12 [OR 1.46 (95%CI 0.81-2.63), *p = *0.2)] and not significantly associated with wheezing at 24 months [OR 1.67 (95%CI 0.95-2.9), *p = *0.07]. Number of sibling alone was not associated with wheezing [OR 1.03 (95%CI 0.85-1.24) *p = *0.73].

**Table 5:** Unadjusted associations between maternal factors and prenatal exposures on the risk of wheezing

	6 months		7 - 12 months		0 - 24 months			
Factor/exposure	Wheezing ever (n = 50/236)		Wheezing ever (n = 59/221)		Wheezing ever* (n = 105/169)		Recurrent wheezing* (n = 39/235)	
	OR (95%CI)	*P *value	OR (95%CI)	*P *value	OR (95%CI)	*P *value	OR (95%CI)	*P *value
Maternal asthma	1.19 (0.38-3.74)	0.76	**2.73 (1.06-7.03)**	**0.03†**	2.35 (0.91-6.06)	0.069	**3.10 (1.10-8.73)**	**0.025¶**
Maternal rhinitis	**2.80 (1.50-5.24)**	**0.001**^**‡**^	1.14 (0.61-2.11)	0.67	**1.73 (1.02-2.93)**	**0.041**	1.78 (0.88-3.58)	0.10
Mother education								
< 5	0.81 (0.33-1.98)	0.65	0.76 (0.31-1.85)	0.54	0.99 (0.48-2.01)	0.98	0.92 (0.33-2.55)	0.88
> 11	1.22 (0.50-2.99)	0.65	1.31 (0.54-3.19)	0.54	1.00 (0.49-2.05)	0.98	1.07 (0.39-2.97)	0.88
Parity								
Primiparous	0.96 (0.52-1.79)	0.91	1.03 (0.57-1.84)	0.91	0.84 (0.51-1.38)	0.49	0.97 (0.48-1.93)	0.93
Type of house								
Wood	0.60 (0.27-1.31)	0.20	0.89 (0.45-1.75)	0.74	0.99 (0.56-1.74)	0.98	0.40 (0.15-1.07)	0.069
Type of floor								
Soil	0.94 (0.37-2.36)	0.90	0.73 (0.31-1.73)	0.48	1.00 (0.48-2.09)	0.98	0.76 (0.28-2.07)	0.59
Tap water								
No	0.64 (0.21-1.93)	0.43	0.35 (0.10-1.20)	0.09	0.70 (0.31-1.55)	0.38	0.59 (0.17-2.04)	0.40
Sewage								
No	1.05 (0.56-1.96)	0.86	0.87 (0.48-1.58)	0.66	1.07 (0.65-1.75)	0.78	0.60 (0.29-1.24)	0.16
Socioeconomic strata								
1 (poorest)	1.10 (0.51-2.35)	0.80	0.59 (0.31-1.14)	0.12	0.76 (0.42-1.36)	0.36	0.92 (0.41-2.07)	0.84
Prenatal exposure to cigarette smoke								
Yes	1.43 (0.77-2.64)	0.24	0.99 (0.55-1.77)	0.98	1.28 (0.78-2.09)	0.32	0.95 (0.48-1.90)	0.89
Exposure to fume wood/coal								
Yes	0.42 (0.12-1.43)	0.16	1.23 (0.52-2.88)	0.63	1.11 (0.52-2.36)	0.77	1.46 (0.55-3.81)	0.43
Exposure to fume trash burning:								
at home	0.95 (0.44-2.04)	0.90	0.87 (0.42-1.81)	0.71	1.05 (0.57-1.92)	0.86	0.67 (0.26-1.69)	0.40
at neighborhood	1.33 (0.71-2.47)	0.36	1.08 (0.61-1.93)	0.77	1.22 (0.75-2.00)	0.41	1.62 (0.80-3.28)	0.17
Pets during pregnancy								
Intradomicilliary (9 mo)	1.01 (0.55-1.87)	0.95	1.23 (0.67-2.23)	0.49	1.12 (0.67-1.88)	0.64	1.12 (0.55-2.28)	0.74
Intradomicilliary (ever)	1.09 (0.58-2.07)	0.77	0.92 (0.52-1.64)	0.79	1.04 (0.64-1.69)	0.87	0.99 (0.50-1.95)	0.98
Dog at home	0.97 (0.52-1.80)	0.93	0.91 (0.51-1.63)	0.77	1.00 (0.61-1.64)	0.98	0.80 (0.40-1.59)	0.53
Cat at home	0.93 (0.36-2.38)	0.89	0.78 (0.30-1.98)	0.60	0.66 (0.30-1.46)	0.31	0.81 (0.26-2.45)	0.71
Poultry/pigs at home	1.39 (0.62-3.15)	0.41	1.64 (0.76-3.55)	0.20	1.43 (0.71-2.89)	0.30	1.49 (0.60-3.68)	0.38
Rodents at home	0.84 (0.38-1.88)	0.68	1.24 (0.54-2.83)	0.60	1.52 (0.75-3.08)	0.23	0.82 (0.33-2.01)	0.67
Cockroaches at home	0.92 (0.44-1.94)	0.84	0.93 (0.46-1.86)	0.83	0.74 (0.41-1.35)	0.33	0.85 (0.38-1.92)	0.70
Gender								
Male	**1.87 (0.99-3.51)**	**0.052**^**§**^	1.37 (0.76-2.45)	0.28	1.61 (0.98-2.64)	0.055	1.47 (0.74-2.93)	0.26

*Cumulative prevalence

†If the true odds ratio for disease in exposed subjects relative to unexposed subjects is (Ψ = 2.73), the test rejects the null hypothesis that this odds ration equals 1 with 0.56 power

Maternal asthma "Yes" (5.4% in non-wheezers vs. 13.6% in wheezers)

¶ If the true odds ratio for disease in exposed subjects relative to unexposed subjects is (Ψ = 3.10), the test rejects the null hypothesis that this odds ration equals 1 with 0.55 power

Maternal asthma "Yes" (5.5% in non-recurrent vs. 15.4% in recurrent wheezers)

‡If the true odds ratio for disease in exposed subjects relative to unexposed subjects is (Ψ = 2.8), the test rejects the null hypothesis that this odds ration equals 1 with 0.89 power

Maternal rhinitis "Yes" (26.3 in non-wheezers vs. 50% in wheezers); (25.4% in non-wheezers vs. 37.1% in wheezers at 24 months)

**§ **If the true odds ratio for disease in exposed subjects relative to unexposed subjects is (Ψ = 1.87), the test rejects the null hypothesis that this odds ration equals 1 with 0.50 power

Male gender "Yes" (48.7% in non-wheezers vs. 64% in wheezers)

There was no association between natural or propane gas and wheezing. In addition, no association was found between wheezing and conditions considered protective, such as poverty and coexistence with pets and poultries. Furthermore, we found no association with well known risk factors, such as cigarette smoking or passive exposure during pregnancy.

Bronchiolitis during the first 6 months was an important risk factor for wheezing at 6 months (aOR 18.3 (95%CI 7.6-44.0), *p *< 0.0001) and 24 months (aOR 20.6 (95%CI 6.08-70.1), *p <*0.0001). Also for recurrent wheezing at 24 months (aOR 6.8 (95%CI 3.0-15.5), *p *< 0.0001), after adjustment for maternal asthma, maternal age, number of siblings and children gender.

## Discussion

Birth cohort studies in different environments and populations are useful to understand the natural history of allergic diseases. Nowadays there are a number of such studies, most of them multicentric, with some common objectives and including a wide range of participants [33-36]. The results, mainly from Europe and US, have shown the importance of particular and common risk factors and their differential influences on allergic diseases, supporting the relevance of doing this type of investigations worldwide, to evaluate the great diversity of settings associated, not only with the climate, geographical location and genetic background, but also with low socio-economic conditions that generate particular risk factors and limit the access to health services. The prevalence of allergic diseases varies among regions and in Latin America, figures of asthma and other allergies in children are very high [37-42]. However, as mentioned, prospective birth cohorts in the region are still scarce, although a growing number are being reported [18-20].

A remarkable finding in our study is the high prevalence of wheezing ever and recurrent wheezing, higher than reported in some European cohorts [43-46], similar to data from the USA [47,48] and lower than those found in some Latin American surveys [19,41]. Considering that 12% of children of this cohort attended a hospital with bronchiolitis, one explanation of our findings is the effect of viral diseases, as has been shown in other studies from Latin America [19]. In fact, bronchiolitis was a high risk factor for wheezing in our cohort, with odds ratio similar to that found when laboratory diagnosis of viral infection was done [49]. Lower respiratory tract infections by Respiratory Syncytial Virus (RSV) are common in tropical developing countries causing 27 to 96% of all acute wheezing hospitalizations in children under 6 month of age [50]. In our children an important proportion of wheezing during this period might be caused by viruses and this related to the finding that only half of children wheezing before 6 months continued wheezing until 24 months. However, the existence of a group with recurrent wheezing (14.2%) suggests that other factors, including atopy, may be influencing this phenotype [18]. RSV infections can induce a change of the Th1/Th2 balance, expressed by an increase of the IL-4/IFN-gamma ratio and a persistent IgE response over the years [51,52], and human rhinovirus-induced bronchiolitis carries a markedly risk of persistent wheezing and childhood asthma [53]. Besides, in the tropics, co-exposure to perennial high concentrations of mite allergens and helminth infections may induce an early allergic response with respiratory effects [54]. Also, living in socially deprived urban areas of underdeveloped countries lead to an early exposure to high levels of endotoxin, in turn associated with increased risk of wheezing during the first year of life [55,56]. Allergy diagnosis by *in vitro *assays and skin prick tests will help to improve phenotype definition in our cohort and obtain data about risk factors for atopy associated-wheezing [57].

In this study, maternal asthma was consistently associated with wheezing and recurrent wheezing in the offspring, a finding already observed in different populations and considered an important risk factor for asthma in children before 5 years [58-61]. The mechanisms of this "maternal effect" and why it is more evident during early childhood are unknown. It has been reported that maternal asthma, as well as the child HLA-G genotype contribute to the presence of bronchial hyperreactivity [62]; in addition, several factors may act in combination with maternal cytokines, antibodies and other mediators that act *in utero *or during the perinatal period [63,64]. Furthermore, recent works in animals suggest that epigenetic mechanisms may play a role [65,66], although the transmission of the maternal susceptibility via epigenetic changes or parental imprinting remains to be demonstrated. Remarkably, this association was detected at 12, from 13 to 24 and 0 to 24 months, suggesting that the maternal effect is on children that start wheezing after 6 months. Moreover, it was also evident with recurrent wheezing, a more severe phenotype and always held up after adjustments for covariates. These results are in agreement with a study in Brazil, were parental asthma was associated with atopic and non-atopic wheezing [67] but in contrast with other where maternal atopy was a risk factor for wheezing in poor-environments [68] because we found no association between maternal sensitization alone and wheezing.

Additionally, and in concordance with other surveys [69-71], we found that male gender was associated with wheezing in cases between 0 and 6 months, but the effect disappeared with increasing age. As it has also been reported, the prevalence and severity of asthma are higher in boys than in girls in the first 10 years of life, but this pattern is reversed after puberty [72]. Although the reasons of this observation remain unknown, there are interesting analysis and hypothesis [73,74].

Contrary to expected, our data show no association of wheezing with active or passive maternal smoking, and the low prevalence of self-reported maternal smoking during pregnancy precluded further association analysis. Therefore, we investigated association between passive intra-domiciliary exposure and wheezing at 6, 12 and 24 months but the results were the same. The prevalence of wheezers was always higher in the group of children with prenatal passive exposure: 6 months (41.1% vs. 50.0%), 12 months (40.4% vs. 47.1%), and 24 months (40.0% vs. 45.2%) but this was not statistically significant. In addition to the type of housing and cultural patterns, another explanation for these results may be a Type II error because the sample is underpowered to detect the effects of smoking. Increasing the sample size and using biological markers of tobacco exposure could help to obtain a more accurate analysis.

The mechanisms of atopic dermatitis are not clear but hereditary factors and environmental exposures are supposed to play important roles [75,76]. A main finding of this study is that in a socially deprived population of the tropics, the natural history of atopic conditions seems to be different to expected according to the "atopic march", where eczema appears before the first two years followed by allergic respiratory symptoms [77,78]. In fact, we found no cases of atopic eczema during the first two years of age, while wheezing was a frequent phenotype. Although we do not know yet if atopy is playing a role in the origin of wheezing in these children, it is clear that early respiratory symptoms predominate over cutaneous, atopy-associated, symptoms. In addition, asthma and allergic rhinitis were the most common allergic manifestations in mothers but there was no eczema among them. In a previous study we found a low frequency of atopic dermatitis in the general population of Colombia [9]. In developed countries, the prevalence of this disease is around 10 to 20% in children under 5 years [79,80], but the ISAAC Phase III study in Latin America reports that, with some exceptions, the prevalence is less than 5% [81]. Under these rates we expected around 15 cases of eczema in our cohort.

Increasing evidence suggest that the "atopic march" includes only a subgroup of patients [82-84]. However, the reasons we found no cases of atopic eczema should be further analyzed. We hypothesized that the genetic background, with an important component of African ancestry and a different distribution of susceptibility alleles for eczema could be protective. Also, particular exposures of these children, with early infections as a hallmark, may confer protection, as has been suggested [56,85]. The diagnostic criteria we used [30] are very important when aiming to study this disease in the tropics because infections and other medical conditions producing "itchy skin" are very frequent. Although cases of eczema may appear in our cohort after 2 years, it has been suggested that a great number begin within the first 12 months, disappearing around 3 years in a significant proportion of children [86]. Therefore, monitoring symptoms of this disorder, as well as other allergic diseases in this cohort is mandatory.

There are reports of associations between low income and asthma [87]. We found no relationship of allergy symptoms with socioeconomic strata, possibly because the study population is homogeneously poor and differences between strata are small. Potential risk factors related to poverty, such as housing conditions, access to tap water and sewage system, exposure to fume from firewood and trash burning, intradomiciliary contact with poultry and pigs and large family size were not associated with wheezing. For most of them the prevalence of exposure among non-affected was high enough to detect an effect. Since the study population is not representative of all the social strata of the city the generalization of these results is limited. Further research is needed to evaluate the potential interactions that lead to allergy phenotypes in socially deprived environments.

Since our study was based in a prospective cohort design that included physician assessment of a broad range of factors, we think that accurate information was obtained. This strategy may reduce the number of participants and requires more resources but it is necessary to increase accuracy avoiding the bias from poorly defined phenotypes, as may occur when the information is obtained only from self-administered questionnaires, especially in populations with low education level. This is especially important for atopic eczema and wheezing because they may not be easily evaluated by parents. Two sources of ascertainment bias should be mentioned: one from the type of housing and living conditions that make it difficult to identify some exposures (e.g. pet ownership, smoke exposure) and other from the detection of phenotypes. The diagnosis of wheezing was made only when patients were evaluated by a physician or had documented history of medications and hospital admissions, as has been recommended [88] and this might bias our attention to the most severe cases, lowering the sensitivity of the survey. However, considering that the biologic samples are intended for serologic and molecular screenings we chose for well-defined phenotypes to reduce heterogeneity. Among the limitations of this study, one is the small number of children recruited, if compared to multicenter surveys. Although, according to the formerly reported prevalence of wheezing in this population our analyses have good power to detect some associations, increasing the number of participants could provide better information. However, many obstacles must be overcome to organize a prospective cohort in countries with high degree of poverty. Low education precludes the use of self-reported questionnaires, limiting the number of enrolled families. Another problem affecting the response rate and the follow-up is the lack of well urbanized neighborhoods, which greatly increase the difficulty of the visits.

As in other birth-cohorts, a valuable selection of biological material has been done in this study [35]. Laboratory tests will help to assess genetic and environmental factors linked to asthma development, providing novel information about disease mechanisms in the tropics. An open question when studying allergies in this zone is the influence of *A. lumbricoides *infection on the process of IgE sensitization to common allergens, especially house dust mites [89]. Thus, IgE serology to *Ascaris spp*. and allergen extracts will be serially performed to explore primary sensitizers and how the infection status influences the frequency and strength of specific IgE response. Another aspect to be evaluated is the relationship between the immunological profile at birth and further wheezing in children. Immune- responsiveness gene expression will be monitored at different time-points. As differences in microbiota composition may influence immune response [90-92], stool samples has been collected from birth to 24 months, to identify gut microbiota composition and explore their potential relationship with the IgE responses and allergies.

## Conclusions

Here we describe the demographic characteristics of the FRAAT birth cohort study, representative of socially deprived urban areas of underdeveloped tropical countries. At 24 months, the history of allergic symptoms is different to the "atopic march" described in some populations of industrialized countries. Although the rates of wheezing were higher than those reported in these countries, cases of eczema were not found. Wheezing is the most frequent phenotype during the first 24 months of life and is strongly associated with maternal asthma. The availability of cord blood serum and other biological samples, will allow to further study risk factors for asthma and atopy during early childhood, including the effects of particular factors related to poverty, such as early microbial and parasite exposure.

## Competing interests

The authors declare that they have no competing interests.

## Authors' contributions

LC conceived the investigation, designed the epidemiological study, supervised the general aspects of the work analyze the data and wrote the manuscript. NA conceived the investigation, designed the epidemiological study, organized and performed the clinical work, managed the acquisition of data, analyze the data and wrote the manuscript. JZ, JS, CQ, AB organize and perform the clinical work, managed databases, collected biological samples and revised the manuscript. AA, MP and KM performed the parasitological examinations and analysis of data and revised the manuscript. D. Mercado collected and processed the house dust samples, analyzed data and revised the manuscript. D. Martinez and SJ collected and processed biological samples for DNA and RNA work, managed databases, analyzed data and revised the manuscript. All authors read and approved the final manuscript.

## Pre-publication history

The pre-publication history for this paper can be accessed here:

http://www.biomedcentral.com/1471-2466/12/13/prepub
